# Dataset on the role of endoglin expression on melanin production in murine melanoma and on the influence of melanin on optical imaging

**DOI:** 10.1016/j.dib.2018.08.110

**Published:** 2018-08-31

**Authors:** Felista L. Tansi, Ronny Rüger, Ansgar M. Kollmeier, Markus Rabenhold, Frank Steiniger, Roland E. Kontermann, Ulf K. Teichgraeber, Alfred Fahr, Ingrid Hilger

**Affiliations:** a Institute of Diagnostic and Interventional Radiology, Experimental Radiology, Jena University Hospital, Am klinikum 1, 07747 Jena, Germany; b Department of Pharmaceutical Technology, Friedrich-Schiller-University Jena, Lessingstrasse 8, 07743 Jena, Germany; c Center for Electron Microscopy, Jena University Hospital, Ziegelmuehlenweg 1, 07743 Jena, Germany; d Institute of Cell Biology and Immunology, University Stuttgart, Allmandring 31, 70569 Stuttgart, Germany

## Abstract

The underlying data demonstrates that the expression of endoglin in murine melanoma cells influences melanin production in the cells. Also, the data shows that melanin production is further increased when the cells are subcutaneously implanted in mice models and that the high melanin production prevents detection of the cells by fluorescence imaging. The processed data presented herein is related to a research article by Tansi et al. (2018) entitled “Endoglin based *in vivo* near-infrared fluorescence imaging of tumor models in mice using activatable liposomes”.

**Specifications Table**

**Table:** 

Subject area	Biology, Chemistry
More specific subject area	Cancer biology and drug delivery
Type of data	Figure, Graphic
How data was acquired	Microscopy, Optical imaging,
Data format	Raw and analyzed
Experimental factors	Murine melanoma cells expressing low or high levels of endoglin were treated with murine-endoglin targeting liposomes (mEnd-IL) which were prepared and used in Tansi et al. [1] then evaluated for fluorescence detection and influence of melanin. Mice subcutaneously implanted with murine melanoma models were intravenously injected with the liposomes and evaluated for melanin production and near-infrared fluorescence detection.
Experimental features	Microscopic and fluorescence optical evaluation of melanin and liposomal fluorescence in endoglin expressing murine melanoma cells or xenografted tumors in mice implanted with the cells.
Data source location	Jena, Germany
Data accessibility	Data is available with this article

**Value of the data**

• Identifying and targeting the molecular markers that influence cancer progression is vital for the management of cancer.
• Overexpression of endoglin by melanoma cells influences melanin production which is associated with an aggressive melanoma phenotype.
• Melanin production by melanoma cells and tumors influences fluorescence imaging negatively.

## 1 Data

The data presented herein comprises optical images of melanoma cells and mice bearing xenografted murine melanomas at different time points after treatment with murine endoglin targeting liposomes (mEnd-IL) and also corresponding graphical data of the fluorescence intensities detected. Furthermore, microscopic images are shown demonstrating the localization of the liposomal fluorescence and melanin in the high endoglin -expressing murine melanoma cells.

## 2 Experimental design, materials and methods

### 2.1 Microscopic detection of melanin and liposomal fluorescence in wild type murine melanoma cells (B16F10-wt) and their stable murine-endoglin transfected counterpart (B16F10mCD105)

**Fig. 1:**
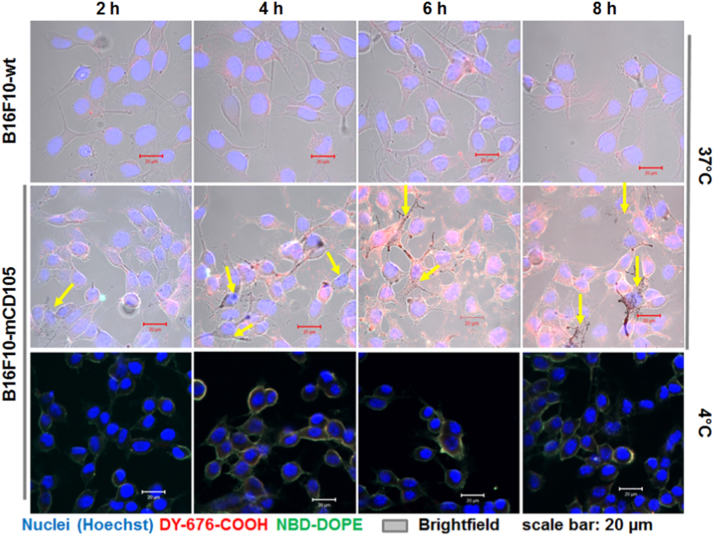
Microscopic detection of mEnd-IL-based fluorescence and melanin in cultured melanoma cells. The microscopic images of monolayer cells reveal mEnd-IL-based fluorescence increase with increasing incubation duration. This fluorescence increase is less peculiar in the B16F10-wt due to low endogenous endoglin expression and higher in the endoglin transfected B16F10mCD105 thanks to increased murine endoglin expression. Melanin is detected as dark spots in cells within the B16F10mCD105 cell population (yellow arrows) and is not clearly visible within the B16F10-wt populations.

The wild type murine melanoma cells (B16F10-wt) with low levels of endogenous endoglin expression, and their stable murine endoglin transfectants (B16F10mCD105) were grown at a density of 30,000 cells/well on poly-L-lysine-coated chambered slides and incubated with 200 nmol (final lipids) of the murine endoglin targeting liposome, mEnd-IL for 2 h, 4 h, 6 h and 8 h at standard culture conditions at 37 °C. To monitor time dependent binding of the liposomes, the B16F10mCD105 were treated with equivalent concentrations of mEnd-IL and incubated at 4 °C for similar durations. Then, the cells were prepared for microscopy as described in [1] and subjected to confocal laser scanning microscopy. The data demonstrates high melanin production and high mEnd-IL-based fluorescence in the melanoma cells stably-expressing endoglin (B16F10mCD105) than the wild type melanoma cells. In the microscopic images, of the B16F10mCD105, melanin is visible as black spots within the cells (Fig. 1, *yellow arrows*). The near-infrared fluorescence signals of the liposomes increase with increase in incubation duration and are higher in B16F10mCD105 than B16F10-wt, due to a higher endoglin expression. Low green fluorescence of the liposomal lipid is seen in cells incubated at 37 °C (Fig. 1, *37 °C*) which can be ascribed to a fast binding, processing and release of the green liposomal phospholipid as demonstrated for other systems [2]. In support of this, liposomal binding at 4 °C is seen to peak already after 4 h to 6 h incubation, as revealed in predominant greenish-yellow signals on the cell membranes of B16F10-mCD105 cells (Fig. 1, *4 °C*).

### 2.2 Optical evaluation of the influence of melanin on liposomal fluorescence detection in wild type murine melanoma cells (B16F10-wt) and their stable murine-endoglin transfected counterpart (B16F10mCD105)

The wild type murine melanoma cells (B16F10-wt), and their stable murine endoglin transfectants (B16F10mCD105) were grown at a density of 2×10^6^ cells and incubated with 200 nmol (final lipids) of the murine endoglin targeting liposome, mEnd-IL for 2 h, 4 h, 6 h and 8 h at standard culture conditions at 37 °C. Thereafter, the cells were washed, pelleted in reagent tubes and subjected to near-infrared fluorescence optical imaging described in [1].

**Fig. 2:**
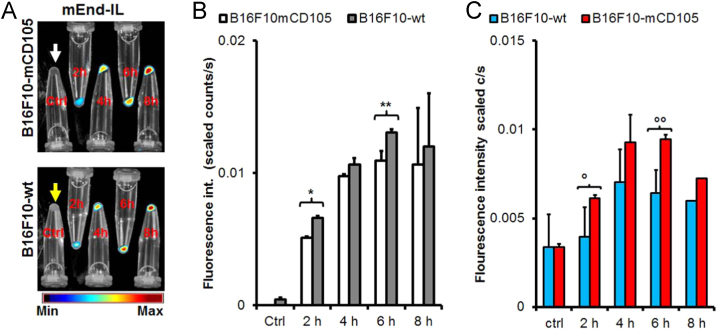
Optical imaging of mEnd-IL based fluorescence and influence of melanin in cultured melanoma cells. A) Near-infrared fluorescence images of cell pellets acquired with the red filter-set (Exc. 615–665 nm, Em. > 700 nm). As seen in the dark color of the cell pellets, (compare B16F10-wt *Ctrl* with B16F10-mCD105 *Ctrl*) the high melanin production in these cells hinders correct detection of the liposomal fluorescence within the cells. B) Corresponding semi-quantitative levels of the near-infrared fluorescence intensities (n = 2/standard deviation) reveal mEnd-IL-based fluorescence increase with increasing incubation duration. This fluorescence increase is significantly higher (*p = 0.009/**p = 0.05) for the B16F10-wt cells than the endoglin transfected, B16F10mCD105 cells at 2 h and 6 h incubation, respectively. C) Semi-quantitative intensities of the green fluorescent liposomal NBD-DOPE acquired with the blue filter-set (Exc. 445–490 nm, Em. > 515 nm) indicate the level of liposomes bound to the cells over time and the level of endoglin expression. At 2 h (*°p=0.06*), and at 6 h (*°°p <0.057*) post incubation of cells with the mEnd-IL, a higher tendency to detect the green fluorescence signal in the stable transfected B16F10-mCD105 cells than in the wildtype cells, was seen despite the interference by the high melanin in the transfected cells. This further indicates that a higher binding of the mEnd-IL to the cells is based on a higher expression of murine endoglin in the B16F10-mCD105 cells.

The data demonstrates that the high endoglin expressing transfectant, B16F10mCD105 when pelleted show a melanin-dependent darkening (Fig. 2, *white arrow*), opposed to the wild type B16F10-wt which appear white when pelleted (Fig. 2, *yellow arrow*). Contrary to the microscopic data which demonstrated a higher mEnd-IL-based fluorescence signal in the endoglin transfected B16F10mCD105 cells, optical imaging shows better fluorescence detection in the wild type melanoma cells. This is due to minimal levels of melanin in the wild type B16F10 cells. In the endoglin transfected B16F10mCD105 cells the increased melanin production that leads to contrast in the pelleted cells interfere with fluorescence detection. Melanin contributes to dispersion of light, thus hindering detection of the high mEnd-IL based NIR fluorescence of the pelleted B16F10mCD105 cells.

### 2.3 in vivo optical evaluation of the influence of melanin on liposomal fluorescence detection in xenografted murine melanoma models

The wild type murine melanoma cells (B16F10-wt), and their stable murine endoglin transfectants (B16F10mCD105) at a density of 1×10^6^ cells/implant were co-implanted subcutaneously in the lower back of immune deficient athymic nude mice as described in [1] and observed for tumor melanin appearance. Also, the mice were intravenously injected with the murine-endoglin targeting liposomes, mEnd-IL and evaluated by optical imaging.

**Fig. 3:**
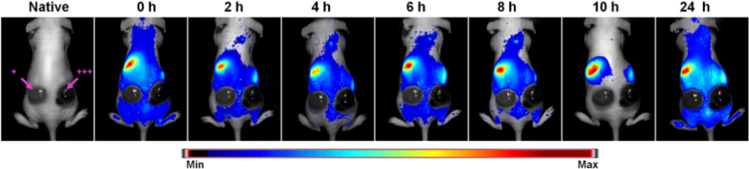
*in vivo* Optical detection of mEnd-IL based fluorescence and melanin in xenografted murine melanoma models. Whole-body NIRF images of mice implanted with B16F10-wt (left flank) and endoglin transfected B16F10-mCD105 (right flank) reveal high melanin production which is seen in the strong black coloration of the tumors (pink arrows). The NIRF images of mice were acquired at the indicated time points post intravenous injection of the mEnd-IL (20 µmol final lipids / Kg body weight). The tumors reveal no fluorescence signals owing to the negative influence of melanin.

The data demonstrates that the wild type B16F10 cells produce xenografts with high melanin production (all 7 cases) in vivo (Fig. 3, *pink arrow +, left flank*). This was similar to the high melanin seen in the endoglin transfected B16F10mCD105-derived xenografts (Fig. 3, *pink arrow +++, right flank*). In support of the interference of fluorescence detection by melanin, both melanoma models were not detectable by fluorescence imaging, as can be seen in the black holes on the positions of the tumors (Fig. 3, 0−24 h). This further substantiates the absorption and dispersion of fluorescence light by melanin and the prevention of light penetration into the tumor tissue.
